# Inequalities in utilisation of general practitioner and specialist services in 9 European countries

**DOI:** 10.1186/1472-6963-11-288

**Published:** 2011-10-31

**Authors:** Irina Stirbu, Anton E Kunst, Andreas Mielck, Johan P Mackenbach

**Affiliations:** 1 Department of Public Health, Erasmus Medical Centre Rotterdam, Erasmus University, The Netherlands; 2 Department of Public Health, Academic Medical Centre (AMC), University of Amsterdam, The Netherlands; 3 Institute of Health Economics and Health Care Management, Helmholtz Zentrum München - German Research Center for Environmental Health, Germany

**Keywords:** health utilization, specialist services, socio-economic inequalities, Europe

## Abstract

**Background:**

The aim of this study is to describe the magnitude of educational inequalities in utilisation of general practitioner (GP) and specialist services in 9 European countries. In addition to West European countries, we have included 3 Eastern European countries: Hungary, Estonia and Latvia. To cover the gap in knowledge we pay a special attention to the magnitude of inequalities among patients with chronic conditions.

**Methods:**

Data on the use of GP and specialist services were derived from national health surveys of Belgium, Estonia, France, Germany, Hungary, Ireland, Latvia, the Netherlands and Norway. For each country and education level we calculated the absolute prevalence and relative inequalities in utilisation of GP and specialist services. In order to account for the need for care, the results were adjusted by the measure of self-assessed health.

**Results:**

People with lower education used GP services equally often in most countries (except Belgium and Germany) compared with those with a higher level of education. At the same time people with a higher education used specialist care services significantly more often in all countries, except in the Netherlands. The general pattern of educational inequalities in utilisation of specialist care was similar for both men and women. Inequalities in utilisation of specialist care were equally large in Eastern European and in Western European countries, except for Latvia where the inequalities were somewhat larger. Similarly, large inequalities were found in the utilisation of specialist care among patients with chronic diseases, diabetes, and hypertension.

**Conclusions:**

We found large inequalities in the utilisation of specialist care. These inequalities were not compensated by utilisation of GP services. Of particular concern is the presence of inequalities among patients with a high need for specialist care, such as those with chronic diseases.

## Background

Access to health care for all in need is a basic social right. At first sight, all European countries have universal insurance coverage and, thus, it is often assumed that these countries also enjoy universal and equitable access to health care services. However, a number of studies indicate that that is not the case[1-7]. Although utilisation of general practitioner (GP) services is distributed fairly equally, independent of income, less well-off people appear to be much less likely to see a specialist than their wealthier counterparts, despite their higher need for such care. This phenomenon is universal in Europe, but seems to be stronger in countries where either private insurance or private practice options are offered[1].

Although a number of international studies have documented inequalities in utilisation of health care services in European countries, this information remains incomplete. Previously only income inequalities in utilisation were studied internationally, thus information is lacking regarding educational inequalities in the use of health services. A theoretical argument in favour of also using education is its growing importance in relation to the relative position of the individual in the distribution of other important assets such as paid labour, occupational status and income level. Additionally, previous studies largely focused on West European countries, missing the growing "new" European populations for which the magnitude of socioeconomic inequalities has hardly been studied[8]. Inequalities in Eastern European countries might be larger than in Western European countries due to recent disruptions in social and health care systems in those countries[8-10]. Finally, all studies on inequalities in utilisation were mainly based on the general population, thus not taking into account people with special needs, such as those with chronic diseases. Large inequalities in the utilisation of health care services in this vulnerable group might indicate specific potential shortcomings within the health care system and support hypotheses about the role of access in explaining differential outcomes of care among people with different socioeconomic status.

The aim of the present study is to describe the magnitude of educational inequalities in utilisation of GP and specialist services in 9 European countries. In addition to West European countries, we have included 3 Eastern European countries: Hungary, Estonia and Latvia. Special attention is also paid to the magnitude of inequalities among patients with chronic conditions.

## Methods

### Data

Data on utilisation of GP and specialist services were derived from micro-level data of national health surveys in 9 European countries (Norway, Ireland, Netherlands, Belgium, Germany, France, Hungary, Estonia, and Latvia). Most surveys were conducted in or after the year 2000, except for the German survey which was conducted in 1998 (Table 1). Sample sizes were above 7000 persons for all surveys, except those from Estonia and Norway. Non-response percentages ranged from about 18% in Ireland up to 42% in the Netherlands and Belgium, while percentages in most other countries were around 30%. Data from 104,503 respondents were included in the analyses.

**Table 1:** Countries included in the analysis and sources of data

Country	Survey name	Year(s)	Non-response (%)	Final sample
Norway	Norwegian Survey of Living Conditions	2002	29.6	6820
Ireland	Living in Ireland Panel Survey	1995, 2002	18.0/22.0*	15051
Netherlands	General social survey (POLS)	2003-2004	41.7 - 38.7	15803
Belgium	Health Interview Survey	1997, 2001	41.5/38.6*	18481
Germany	German National Health Examination and Interview Survey	1998	38.6	7124
France	Health, Health Care and Insurance Survey (IRDES)	2004	30.0*	17828
Hungary	National Health Interview Survey Hungary	2000, 2003	21.0 - 28.0	10532
Estonia	Health Behavior among Estonian Adult Population	2002, 2004	33.0/38.0*	4376
Latvia	Finbalt Health Monitor	1998; 2000; 2002; 2004	20.0 - 40.0	8488
Europe				104503

* Percentage non-response households

In all surveys, utilisation of GP and specialist services was self-reported. All participants were asked how many times they visited a GP or a specialist in a specified period of time. In all countries the recall period for utilisation of GP and specialist services was 12 months, except for the Netherlands and Belgium where the recall period was only 2 months.

In order to take the need for care into account we have included the measure of self-assessed health. Self-assessed health was rated according to 5 answer categories from the healthiest to the least healthy. The exact answer categories ranged in most countries from "very good" to "very bad", although there were some variations between countries. Additionally, the utilisation of services was investigated among people with chronic diseases. In all surveys the presence of chronic diseases was self-reported, except for Ireland that had no data on chronic diseases. Because each survey varied depending on the type and number of chronic diseases included, we selected only those chronic disease that were present in at least 6 of the 9 surveys: angina pectoris, arthritis, asthma, bronchitis, cancer, diabetes, hypertension, myocardial infarction, stroke, and ulcers. A patient was considered to be having a chronic disease if s/he reported having at least one from the above mentioned chronic conditions. Information on diabetes and hypertension was included in all surveys, and prevalence rates were high in all countries; this allowed us to use these diseases for a more in-depth analysis.

Socioeconomic position was measured using the level of education, which represents the highest level of completed education of the respondent. The level of education was initially classified according to national categories, which were subsequently reclassified into three levels of the International System of Classification of Educations (ISCED): primary or no education and lower secondary education; higher secondary education; tertiary education.

### Analysis

First, we assessed educational inequalities in utilisation of GP and specialist services using prevalence rates of having made at least one visit to a GP or specialist. Prevalence rates were calculated for each type of service by education group and participating country. The prevalence rates were standardized by 5-year age groups and gender to the total survey population, as a representative sample for the standard European population. We use prevalence rates to judge about the absolute differences of GP or specialist services use between different educational groups.

Second, we estimated relative inequalities in utilisation of GP and specialist services among higher and lower educational groups of the general population using the relative index of inequality (RII). The RII is a regression-based index used to measure socioeconomic inequalities in health in a comparable way in different countries[11,12]. The RII quantifies the relative position of each educational group within the hierarchy of all educational groups. This rank measure is related to health indicators by means of log-binomial regression. The RII results in a ratio that can be described as the prevalence ratio of preventive services utilisation at the very bottom of the educational hierarchy compared to the very top of the hierarchy.

Third, we estimated relative inequalities by education in utilisation of GP and specialist services among persons with chronic diseases, hypertension and diabetes.

All calculations were done using log-binomial regression analysis in SAS statistical package (version 8.02). We included categorical variables in the regression models, representing 5-year age groups and gender, to control for demographic confounders. To take the need for care into account, we adjusted our results by the ranked measure of self-assessed health, which quantifies the relative position of each group of people in one answer category in the hierarchy of all answer categories. Ranked measure of self-assessed health was calculated on the basis of the cumulative relative frequencies of the valid cases and allows for better comparison between countries.

## Results

The study populations in the different European countries did not differ greatly regarding age and gender distribution (Table 2), except for the Baltic countries where there were slightly more younger female respondents. In contrast, there was a considerable difference in educational distribution between the countries, with Norway and the Netherlands having fewer people with lower education, and Germany, Hungary and Ireland having fewer people with higher education. In most countries, the percentage of people reporting visiting a GP ranged from 67% to 80%; it was substantially lower in Latvia, the Netherlands and Belgium (range 35% to 46%). In the latter 2 countries the lower rates of GP visits is probably related to the shorter recall period (2 and 3 months, respectively, versus 12 months in all other countries). The highest report for visiting a specialist was in Germany (75%) and the lowest was in Norway (17%).

**Table 2:** Background information on the study populations.

Country	Age above 50 yrs (%)	Gender distribution (% men)	% Lower secondary education and below	% Upper secondary education	% Tertiary education	People reporting visiting a GP (%)	People reporting visiting a specialist (%)
Norway	39.8	50.0	17.5	56.6	25.9	74.8	17.0
Ireland	37.5	49.5	55.9	29.8	14.3	72.8	24.8
Netherlands	42.7	48.5	39.3	37.7	22.9	35.6	18.0
Belgium	42.0	48.5	41.0	30.0	29.0	46.8	22.9
Germany	42.7	48.4	43.0	43.1	13.9	67.9	74.7
France	39.5	49.1	53.7	18.9	27.4	80.5	56.9
Hungary	42.8	44.6	57.6	29.0	13.4	74.1	51.7
Estonia	30.1	42.3	47.9	34.5	17.6	67.3	44.6
Latvia	28.9	43.5	44.3	34.6	21.1	44.5	29.1
Europe	39.4	47.8	46.0	32.3	21.7	59.2	35.7

Only in Belgium and Germany were lower educated people significantly more likely to report a visit to a GP (RII is 1.29 and 1.20, respectively; Table 3). After adjustment for self-assessed health the RII slightly decreased in all countries. Although utilisation of GP care was fairly equally distributed between educational groups, there was a general tendency of lower use by the lower educated (RIIs just below 1 in all countries except Belgium and Germany). In Belgium and Germany significantly higher utilisation of GP services by lower educated groups remained, although weakened. On the other hand, after adjustment for self-assessed health, in Hungary higher educated people used GP services significantly more often compared to the lower educated group (RII = 0.87 CI: 0.80-0.95).

**Table 3:** Prevalence rate (PR) and Relative index of inequality (RII) in utilisation of GP and specialist services

Country	PR ^a ^Lower secondary education and below	PR Upper secondary education	PR Tertiary education	**RII (95%CI)^b^**, Men & women, Adjusted for age & gender	**RII (95%CI)** Men & women Adjusted for age, gender & SAH^c^	**RII (95%CI)**, Men Adjusted for age & SAH^c^	**RII (95%CI)**, Women Adjusted for age & SAH^c^
*Utilization of GP services*							
Norway	75.1	75.8	73.3	1.07 (0.96-1.20)	0.98 (0.87-1.10)	1.06 (0.90-1.26)	0.92 (0.78-1.08)
Ireland	74.7	69.7	71.3	1.07 (0.95-1.20)	0.97 (0.87-1.09)	0.87 (0.74-1.04)	1.07 (0.92-1.26)
Netherlands	35.6	35.8	33.6	1.08 (0.98-1.20)	0.93 (0.84-1.04)	0.96 (0.81-1.14)	0.98 (0.84-1.13)
Belgium	52.1	44.3	39.1	1.29 (1.19-1.40)	1.13 (1.03-1.23)	1.10 (0.96-1.25)	1.17 (1.04-1.32)
Germany	70.6	71.4	62.1	1.20 (1.07-1.34)	1.16 (1.04-1.30)	1.22 (1.04-1.42)	1.10 (0.93-1.30)
France	79.7	81.5	81.0	0.98 (0.91-1.06)	0.93 (0.86-1.01)	0.89 (0.79-1.01)	0.99 (0.88-1.10)
Hungary	73.2	73.1	74.8	0.97 (0.89-1.06)	0.87 (0.80-0.95)	0.82 (0.72-0.94)	0.92 (0.82-1.03)
Estonia	69.0	70.0	67.2	0.98 (0.85-1.13)	0.91 (0.79-1.05)	0.82 (0.65-1.03)	0.97 (0.81-1.18)
Latvia	47.3	45.3	46.8	0.97 (0.85-1.10)	0.92 (0.80-1.04)	1.00 (0.81-1.23)	0.87 (0.74-1.03)
Europe	62.4	61.4	58.9	1.09 (1.06-1.13)	1.00 (0.97-1.04)	1.00 (0.95-1.05)	1.02 (0.97-1.07)
							
*Utilization of specialist services*							
Norway	14.0	17.7	18.5	0.72 (0.57-0.91)	0.61 (0.48-0.78)	0.66 (0.46-0.96)	0.59 (0.43-0.81)
Ireland	25.6	22.5	27.1	0.84 (0.70-1.02)	0.59 (0.49-0.72)	0.57 (0.43-0.76)	0.62 (0.47-0.80)
Netherlands	17.9	18.1	17.2	1.05 (0.91-1.21)	0.86 (0.73-1.00)	0.90 (0.72-1.13)	0.88 (0.71-1.09)
Belgium	20.8	22.1	25.5	0.73 (0.65-0.82)	0.59 (0.52-0.67)	0.73 (0.59-0.89)	0.54 (0.46-0.64)
Germany	74.2	78.6	76.2	0.90 (0.80-1.00)	0.87(0.78-0.97)	0.93 (0.80-1.09)	0.86 (0.74-1.00)
France	51.9	58.7	66.5	0.60 (0.55-0.66)	0.55 (0.51-0.61)	0.55 (0.47-0.64)	0.59 (0.52-0.66)
Hungary	47.4	54.0	59.6	0.72 (0.65-0.80)	0.58 (0.52-0.65)	0.60 (0.51-0.72)	0.61 (0.53-0.70)
Estonia	42.5	45.6	51.4	0.76 (0.64-0.91)	0.68 (0.57-0.81)	0.62 (0.46-0.84)	0.72 (0.57-0.90)
Latvia	26.0	29.0	39.3	0.51 (0.44-0.60)	0.47 (0.40-0.55)	0.51 (0.38-0.68)	0.46 (0.38-0.55)
Europe	33.1	36.4	40.2	0.74 (0.71-0.77)	0.65 (0.62-0.67)	0.70 (0.65-0.75)	0.66 (0.62-0.69)

^a ^Prevalence rate per 100 persons (men and women combined), age and gender standardized to the total survey population;

**^b ^**Relative index of inequality and 95% confidence interval adjusted for age and gender

**^c ^**SAH = Self-assessed health

The prevalence of specialist services use was more diverse compared with GP services, with higher utilisation in Germany, France, Hungary and Estonia (above 40% for both higher and lower educated groups; Table 3). Higher educated people reported using specialist services significantly more often than lower educated people in almost all countries, except for the Netherlands (RII = 1.05) where utilisation was equal for higher and lower educated groups. After adjustment for self-assessed health, people with higher education reported using specialist services significantly more often in all countries, without exceptions. Relative inequalities were smaller in the Netherlands and Germany (RIIs around 0.86) and were very pronounced in Latvia (RII = 0.47).

The pattern of utilisation of GP and specialist services for patients with chronic diseases, diabetes and hypertension was similar to that of the general population: lower and higher educated persons with chronic diseases were equally likely to use GP services in most countries (Table 4). Only in Belgium and Germany did lower educated patients report using GP services slightly more often. On the other hand, higher educated patients with chronic conditions used specialist services significantly more often than lower educated patients (RII = 0.87 and lower), except in the Netherlands (RII = 0.92; Table 4). These inequalities tended to be larger in Norway, Belgium, France, Hungary and Latvia, and were somewhat smaller in the other countries.

**Table 4:** Relative index of inequality (RII) in utilisation of GP and specialist services among patients with chronic diseases; men and women combined.

Country	Chronic diseases	Diabetes	Hypertension
	RII ^a ^(95% CI)	RII (95% CI)	RII (95% CI)
*Utilization of GP services*			
Norway	0.99 (0.79-1.24)	0.99 (0.57-1.69)	0.96 (0.71-1.31)
Netherlands	0.92 (0.78-1.08)	0.71 (0.47-1.07)	0.97 (0.77-1.23)
Belgium	1.15 (1.00-1.31)	1.27 (0.86-1.88)	1.19 (0.98-1.44)
Germany	1.11 (0.94-1.32)	1.74 (1.02-2.97)	1.14 (0.90-1.44)
France	1.03 (0.89-1.19)	0.98 (0.66-1.46)	1.03 (0.86-1.24)
Hungary	0.91 (0.78-1.06)	0.93 (0.69-1.24)	0.90 (0.74-1.10)
Estonia	0.99 (0.81-1.22)	1.10 (0.61-1.98)	1.00 (0.75-1.33)
Latvia	0.97 (0.75-1.25)	0.71 (0.31-1.67)	1.04 (0.75-1.44)
Europe	1.03 (0.97-1.09)	1.03 (0.88-1.20)	1.05 (0.96-1.14)
			
*Utilization of specialist services*			
Norway	0.55 (0.36-0.84)	0.62 (0.25-1.57)	0.50 (0.28-0.90)
Netherlands	0.92 (0.74-1.13)	0.71 (0.43-1.18)	0.86 (0.62-1.18)
Belgium	0.64 (0.52-0.78)	0.50 (0.29-0.87)	0.65 (0.48-0.87)
Germany	0.87 (0.74-1.02)	0.83 (0.52-1.34)	0.87 (0.69-1.09)
France	0.68 (0.54-0.85)	0.77 (0.49-1.20)	0.64 (0.51-0.79)
Hungary	0.63 (0.52-0.75)	0.72 (0.52-1.00)	0.60 (0.47-0.77)
Estonia	0.76 (0.59-0.97)	0.77 (0.38-1.57)	0.74 (0.51-1.07)
Latvia	0.60 (0.43-0.84)	0.83 (0.25-2.70)	0.66 (0.43-1.01)
Europe	0.71 (0.66-0.77)	0.72 (0.59-0.86)	0.69 (0.62-0.77)

**^a ^**Relative index of inequality (95% confidence interval) adjusted for age, gender, and self-assessed health

## Discussion

People with a lower education level used GP services slightly less often as those with a higher level of education in most countries (except for Belgium and Germany). At the same time, higher educated people used specialist care services significantly more often in all countries (except for the Netherlands). Educational inequalities in utilisation of specialist care among women were slightly larger than among men in some countries, although the general pattern of use was similar for both men and women. Inequalities in utilisation of specialist care were equally large in Eastern European and in Western European countries, except for Latvia where the level of inequalities was somewhat larger. Similarly large was the level of inequalities in utilisation of specialist care among patients with chronic diseases, diabetes, and hypertension.

The high percentage of non-response in some countries could have biased our results if both the educational level and the reported utilisation of services had been unequally distributed among respondents and non-respondents. Although some studies reported that non-response is related to socioeconomic status[13-15], previous evaluations showed that the association between utilisation of services and socioeconomic status would not greatly change if non-respondents were included with respondents[16,17]. Nevertheless, in the present study we cannot exclude the possibility that an over-representation of sicker lower educated people in the non-response group may have led to some underestimation of the pro-rich inequalities in prevalence rates of utilisation reported here.

We used education as an indicator of socioeconomic position. Education allows the classification of individuals who do not work, prevents reverse causation, and facilitates international comparisons due to its relative ease of measurement. In addition, recent studies suggest that in some countries education has an independent effect and is more strongly related to the likelihood of health services utilisation than income and employment status[18,19]. On the other hand, educational level might not accurately indicate an older person's current socioeconomic position since it is acquired early in life and may inadequately reflect changes in socioeconomic position during adult life[20].

There were large differences between countries in the educational distribution. These differences reflect, in part, the real situation of educational attainment in different countries of Europe[21]. However, there is a possibility that the ISCED classification is not flexible enough to accommodate different national schemes. To cope with the differences in educational classification we used the RII, a measure that takes educational distribution into account[11,12]. Additionally, RII has the advantage that it can be applied in a comparable way to all countries provided that the educational classifications are strictly hierarchical.

The recall period for use of GP and specialist services was shorter in the Netherlands and Belgium than in the other countries. A longer recall period would have influenced the overall utilisation rates for the total population. It is, however, unlikely that it would have a differential effect on utilisation of services by different educational groups.

Self-assessed health was used in order to control for the health care needs of the population. Although the measure of self-assessed health is often used in health care research due to its wide availability and good comparability, it does not completely encompass the full spectrum of need. A better control for need would likely result in greater inequalities in specialist visits, while inequalities in GP visits might have also emerged in some countries.

Most European countries have achieved universal access to health care. However, the results of the present study show that universal access does not mean equal use. One might argue that differences in utilisation do not directly reflect inequalities in access to care. The decision to use health care services and the type of provider is, after all, a personal choice. Nevertheless, this personal choice is affected to a large extent by various enabling and predisposing factors. People from lower socioeconomic strata are likely to have fewer enabling factors and more barriers to use specialist care.

European countries have very different health care systems. For example, some countries operate with GP gate keeping (e.g. the UK, the Netherlands), others have more direct access to specialists and hospital care (France); some countries use only public insurance (Germany, the Netherlands), others only private or a combination of the two (Spain, Portugal); some countries use co-payments, others do not; etc. Regardless of the way the system is organised, we find a generalised pattern of differential access to primary and secondary care for people with different socioeconomic positions. Such a universal pattern indicates that patients with a lower socioeconomic position encounter barriers that are common in all countries, and thus lie beyond the national structure and organisation of the health care system.

Proper communication between the patient and health provider where the patient not only receives information about his disease, diagnostic procedures, and treatment, but also feels understood and helped is essential. Successful communication contributes to both patient outcomes[22,23] and general satisfaction with services[24,25]. People with a lower socioeconomic position may better appreciate communication with the GP than with a specialist, as the former may be clearer in discussing the disease, be better at understanding and addressing the needs of the patient and, thus, be perceived as more trustworthy. On the other hand, patients with a higher socioeconomic position may trust a "higher specialised" provider and request contact with the specialist, or seek this contact directly thus avoiding the primary care provider. It is suggested that patients with lower education, lower income and ethnic background express more preference to see a GP for their initial care than better educated, higher income white patients[26], although research in this area is very limited and sometimes contradictory[27]. Higher educated patients that chose a GP for their initial contact (either as personal choice or due to organisational enforcement, as in countries with a gate keeping system) are usually better able to articulate their needs for the specialist and have greater assertiveness regarding being referred to one[28,29], leading to a higher number of referrals.

One may suggest that a simple substitution of care occurs i.e. equal quality care for the same problem, which is performed by one type of provider instead of another without any consequences for the health outcomes of the patient. Our data, however, indicate that lower-educated people use GP services slightly less often compared to higher-educated people in most European countries, while inequalities in the use of specialists are large. A better control for need of care may even reveal pro-rich inequalities in the use of GP services. Thus, we do not find evidence for the substitution of care. Others also showed that the likelihood to consult a specialist increases given a consultation with the general practitioner[2].

Another common feature of the health care system is its enormous complexity: whichever type of organisation exists in a country it is never easily understood, particularly by those with a lower socioeconomic position. This complexity is often coupled with constant changes in the way the system operates that may disorient even well-educated patients. Since primary care (GP practices) is the easiest, most accessible and least changeable type of care, people with a lower socioeconomic position may not feel inclined to go further up the hierarchy of the health care organization, in order to avoid this confusing complexity.

Within the generalised pattern of differential utilisation of different types of services, there remain some variations that indicate that national health care systems may play an additional role in (dis-)motivating patients to use particular types of care. For example, compared to other countries, we observed larger inequalities in the use of specialist care in Latvia and smaller inequalities in the Netherlands. Similar differences were also observed in studies on income inequalities in utilisation of care[30]. It is plausible that these variations in the magnitude of inequalities are driven by differences in health system characteristics, such as sources of finance and service delivery practices. For example, in the Netherlands there is a stronger GP gate keeping system compared to other countries included in this study. A strong GP gate keeping system may allow a better control of the patient flow to specialists that is in accordance with clinical guidelines (and needs of the patients), thus leaving less room for inequalities in the utilisation of specialist care to occur compared to a more free-way system[31].

We hypothesized that inequalities in access to care in East European countries would be larger than in the West European countries due to disruption of the social protection and health care systems that occurred during the 1990s in many former Soviet countries. Our data do indicate larger inequalities in use of specialist care in Latvia. Compared to the neighbouring countries, Latvia has implemented a system with larger co-payment mechanisms for public health services. Thus, the financial barriers met by the population for the use of health services might have resulted in much lower utilisation rates and the highest level of inequalities observed in the present study. Also in Hungary, in addition to large inequalities in utilisation of specialist care, there were significant pro-rich inequalities in the use of GP services, indicating gross general inequalities in utilisation of health services. Our findings are supported by studies reporting larger inequalities in mortality amenable to medical care found in East European countries compared to West European countries [32-34]. However, in Estonia the magnitude of inequalities in utilisation of care was similar to that of West European countries, which indicates that the problem is limited to particular countries and can not be generalised to all East European countries.

The present study paid particular attention to people with chronic diseases. The results show large inequalities in utilisation of specialist services in this vulnerable group. Hampered access to specialist care might have a more severe impact on the health status of patients with high need, such as the chronically diseased, compared to the general population. Thus, there is an urgent need to investigate and remove barriers to the use of specialist care among patients with chronic diseases.

## Conclusions

In summary, large inequalities were observed in the utilisation of specialist care that are not compensated for by the use of GP services. Of particular concern is the presence of inequalities among patients with a high need for specialist care, such as those with chronic diseases, which raises important issues regarding the access to care among vulnerable subgroups.

## Competing interests

The authors declare that they have no competing interests.

## Authors' contributions

IS designed the study, performed the statistical analysis and wrote the main draft of the manuscript. AK supervised the study and provided comments to all drafts of the manuscript. AM contributed to study design and provided comments to drafts of the paper. JM conceived the study and contributed to draft the manuscript. All authors read and approved the final manuscript.

## Pre-publication history

The pre-publication history for this paper can be accessed here:

http://www.biomedcentral.com/1472-6963/11/288/prepub
